# Characterization of the *Burkholderia mallei tonB* Mutant and Its Potential as a Backbone Strain for Vaccine Development

**DOI:** 10.1371/journal.pntd.0003863

**Published:** 2015-06-26

**Authors:** Tiffany M. Mott, Sudhamathi Vijayakumar, Elena Sbrana, Janice J. Endsley, Alfredo G. Torres

**Affiliations:** 1 Department of Microbiology and Immunology, University of Texas Medical Branch, Galveston, Texas, United States of America; 2 Department of Pathology, University of Texas Medical Branch, Galveston, Texas, United States of America; 3 Sealy Center for Vaccine Development, University of Texas Medical Branch, Galveston, Texas, United States of America; George Washington University, UNITED STATES

## Abstract

**Background:**

In this study, a *Burkholderia mallei tonB* mutant (TMM001) deficient in iron acquisition was constructed, characterized, and evaluated for its protective properties in acute inhalational infection models of murine glanders and melioidosis.

**Methodology/Principal Findings:**

Compared to the wild-type, TMM001 exhibits slower growth kinetics, siderophore hyper-secretion and the inability to utilize heme-containing proteins as iron sources. A series of animal challenge studies showed an inverse correlation between the percentage of survival in BALB/c mice and iron-dependent TMM001 growth. Upon evaluation of TMM001 as a potential protective strain against infection, we found 100% survival following *B*. *mallei* CSM001 challenge of mice previously receiving 1.5 x 10^4^ CFU of TMM001. At 21 days post-immunization, TMM001-treated animals showed significantly higher levels of *B*. *mallei*-specific IgG1, IgG2a and IgM when compared to PBS-treated controls. At 48 h post-challenge, PBS-treated controls exhibited higher levels of serum inflammatory cytokines and more severe pathological damage to target organs compared to animals receiving TMM001. In a cross-protection study of acute inhalational melioidosis with *B*. *pseudomallei*, TMM001-treated mice were significantly protected. While wild type was cleared in all *B*. *mallei* challenge studies, mice failed to clear TMM001.

**Conclusions/Significance:**

Although further work is needed to prevent chronic infection by TMM001 while maintaining immunogenicity, our attenuated strain demonstrates great potential as a backbone strain for future vaccine development against both glanders and melioidosis.

## Author Summary

*Burkholderia mallei* and *B*. *pseudomallei* are the causative agents of glanders and melioidosis, respectively. In addition to the recent rise in cases of glanders and the endemicity of melioidosis worldwide, these pathogens have gained attention as potential bioweapons. Further, these pathogens have huge potential for aerosol delivery and often produce fatal infection amongst untreated individuals. Both pathogens are difficult to treat, and even with antibiotic intervention, patients relapse or get re-infected. A big challenge for vaccine development against these pathogens includes identification of broadly protective antigens and a better understanding of the correlates of protection from both acute and chronic infections. Our study is the first to demonstrate significant protection against a lethal challenge with both *Burkholderia* species. Because TMM001 persists in immunized mice, we propose that this attenuated organism is a promising backbone-based strain from which a legitimate vaccine candidate can be generated.

## Introduction

Melioidosis and glanders are severe zoonotic diseases caused by two closely related Gram-negative pathogens known as *Burkholderia pseudomallei* and *B*. *mallei*, respectively [1,2]. The genomic relatedness between these two pathogens suggests that *B*. *mallei* is a host-adapted clone of *B*. *pseudomallei*, which evolved from a process of reductive evolution. Genes retained by *B*. *mallei* share 99% sequence identity with their *B*. *pseudomallei* orthologs and of those, 650 genes have been identified as putative virulence determinants via *in silico* genomic subtraction from non-pathogenic *Burkholderia* species [3]. In addition, the presence of very few *B*. *mallei* specific genes suggest it’s possible to generate a live attenuated vaccine with a *B*. *mallei* backbone that can cross-protect against both melioidosis and glanders [4].

Where *B*. *pseudomallei* is an environmental saprophytic pathogen ubiquitous in soil and fresh water surfaces, *B*. *mallei* is an obligate mammalian pathogen that typically infects solipeds (horses, donkeys, etc) [1,5]. Despite epidemiological differences, the clinical and pathological manifestations of *B*. *pseudomallei* or *B*. *mallei* infections bear striking resemblance. Both pathogens can be contracted via the cutaneous, oral and/or inhalational routes. Depending on the dose and route of transmission, *B*. *pseudomallei* or *B*. *mallei* infection may result in an acute or chronic disease. Clinical manifestations of acute infection from either disease, which include fever, malaise, abscess formation, pneumonia and sepsis, are non-specific. The lack of pathognomonic symptoms, in addition to their ability to cause silent infection, makes rapid and accurate diagnosis problematic for these *Burkholderia* infections. Since mortality rates among severe infections are high, and there are no reliable antibiotic therapy or licensed pre- and post-exposure vaccines, both pathogens remain top candidates for bioterrorist use and thus have been classified as category B, tier 1, biothreat agents [1]. The destructive potential of *B*. *pseudomallei* and *B*. *mallei* has heightened concerns among public health officials due to the increased potential of opportunistic infection among growing populations of diabetic and immunocompromised people [2]. For military personnel and susceptible individuals, the availability of a vaccine would be the most efficacious and cost-effective way to protect from disease.

Progress in vaccine development shows formulations consisting of subunits or live-attenuated strains are the most effective in conferring protection against both pathogens. Subunit vaccines consisting of purified protein [6]; recombinant Hcp proteins [7]; lipopolysaccharide (LPS) [8,9]; truncated recombinant proteins LolC and PotF [10]; and outer membrane vesicles (OMV) [11] have achieved the greatest protection to date. While encouraging, subunit vaccines provided only partial protection, which is attributed to their inability to generate broad protective immunity, specifically cell mediated immunity [12].

Live attenuated vaccines are recognized for their ability to elicit strong broad immune responses that provide long-lasting protection [12]. Thus far, attenuated mutants lacking a functional *purN*, *purM*, *aroB*, *ilvl*, or *bipD* genes in *B*. *pseudomallei*, and *ilvl* or DD3008 (capsule) genes in *B*. *mallei* have been evaluated for their protective potential [12]. Although these candidates have proven capable of providing significant protection during the acute stage of infection, none have yet to afford full protection during the chronic stage of infection.

To create a live attenuated *B*. *mallei* mutant that will generate a protective immune response against chronic infection, we focused on iron transport systems as a target of mutagenesis. For a majority of bacterial pathogens, the acquisition of iron and iron complexes has long been recognized as major pathogenic determinant and thus also represent a promising target for vaccine development. In the host environment, free iron is too scarce and iron complexes are too large to diffuse effectively through porin channels. To survive in these growth-limiting conditions, bacteria utilize siderophores and/or high-affinity outer-membrane receptors to uptake iron and iron complexes [13]. In the case of *B*. *pseudomallei* and *B*. *mallei*, very little information exists concerning iron uptake mechanisms in the host and their roles in virulence. In one study, Kvitko el at., generated single, double and quadruple *B*. *pseudomallei* mutants defective in siderophores and/or hemoglobin utilization [14]. While mutants defective in these systems are often attenuated, the *B*. *pseudomallei* mutants remained fully virulent in a murine model of acute melioidosis [14]. Failure to eliminate virulence was attributed to redundancy in the iron transport system, citing a reliance on alternative iron sources and acquisition mechanisms.

To negate this redundancy, we targeted the inner membrane energy transfer protein TonB, an essential component that interacts with all outer membrane receptor proteins that carry out high-affinity binding and energy dependent iron uptake [15,16,17]. When assessed in multiple models of infection, *tonB* mutants displayed severe attenuation compared to their wild-type homologs [18,19,20,21]. In the case of *K*. *pneumoniae*, Hsieh *et al*. showed 100% protection in challenge mice previously vaccinated with the *tonB* mutant homolog [20]. Thus, *Burkholderia* TonB-dependent iron-transport systems, specifically their contribution to survival, persistence and potential as targets for attenuation, should be investigated further. In this communication, we describe the construction and characterization of a *B*. *mallei tonB* mutant as a backbone strain for subsequent vaccine development against acute inhalational murine glanders and melioidosis.

## Methods

All manipulations of *B*. *mallei* were conducted in CDC/USDA-approved and registered biosafety level 3 (BSL3) facilities at the University of Texas Medical Branch (UTMB), and experiments with select agents were performed in accordance with BSL3 standard operating practices. The animal studies were carried out in strict accordance with the recommendations in the Guide for the Care and Use of Laboratory Animals of the National Institutes of Health. The protocol (IACUC #0503014B) was approved by the Animal Care and Use Committee of the UTMB.

### Bacterial Strains and Growth Conditions

The bacterial strains and plasmids used in this study are listed in Table 1. All *E*. *coli* strains were grown in Luria-Bertani (LB) media at 37°C or 30°C, as required. For all the experiments, all *B*. *mallei* strains were taken from freezer stocks, plated on LB agar containing 4% glucose (LBG), and incubated at 37°C for 3 days. For liquid cultures, a few colonies (2–3) were inoculated into 20 mL of LBG broth and incubated overnight with agitation at 37°C. When employing antibiotic selection, we used kanamycin and polymyxin B at concentrations of 50 μg/mL and 30 μg/mL, respectively. For counter-selection, co-integrates were grown in YT broth (10 g of tryptone and 10 g of yeast extract) and then plated on sucrose agar (YT agar supplemented with 5% sucrose), as described by Hamad et al. [22]. When appropriate, LBG broth and agar were supplemented with FeSO_4_ at a concentration of 200 μM. Unless otherwise stated, wild type *B*. *mallei* ATCC 23344 or CSM001 (*B*. *mallei* Lux), *B*. *mallei* TMM001 (*tonB* mutant), and TMM002 (pTonB-comp) were used in all experiments.

**Table 1:** Bacterial strains and plasmids.

Strains	Relevant Features	Reference
*B*. *mallei* ATCC 23344	Human clinical isolate; Km^S^Pb^R^	[54]
*B*. *pseudomallei* K96243	Human clinical isolate; Km^R^Gm^R^Zeo^R^ Pb^R^	[55]
*B*. *mallei* CSM001	*B*. *mallei* ATCC 23344 with a mini-Tn*5*::*lux*Km_2_; Km^R^Pb^R^	[25]
*B*. *mallei* TMM001	*B*. *mallei* ATCC 23344 with an unmarked deletion of *bmaa1801 (*∆*tonB*)	This study
*B*. *mallei* TMM002	∆*tonB* complemented with pTonB-comp; Km^R^	This study
*E*. *coli* S17-1	For conjugal transfer, *recA thi pro hsdRM*+ RP4:2-Tc:Mu:KmTn7 Tp^R^ Sm^R^	[56]
*E*. *coli* S17-1(pTonB-allex)	*E*. *coli* S17.1 with the recombinant suicide plasmid pTonB-allex; Km^R^	This study
**Plasmids**		
pMo130	Suicide vector for allelic exchange in *Burkholderia*; used to construct pTonB-allex; pUC19 *ori*, RK2 *ori*T, *xylE*, *sacB*, Km^R^	[22]
pTonB-allex	pMo130 derived recombinant suicide plasmid used to generate ∆*tonB*; Km^R^	This study
pMo168	Replicative vector for *Burkholderia; or*ipBBR1, *mob*+, *xylE*, Km^R^	[22]
pTonB-comp	pMo168 derived recombinant replicative vector used to complement ∆*tonB*; pMo168:: *bmaa1801;* Km^R^	This study

### DNA, PCR and Cloning Methods

Cloning methods were performed as previously described [22]. Chromosomal and plasmid DNA were isolated by using the DNeasy Qiagen Blood and Tissue kit, and the QIAGEN Plasmid Mini Kit, respectively (Qiagen, Inc., Valencia, CA). Polymerase chain reaction (PCR) products were purified with either the QIAquick PCR purification kit or QIAquick gel extraction kit (Qiagen). Restriction enzymes and T4 DNA ligase were purchased from NEB and used in accordance with the manufacturers’ instructions (New England Biolabs Inc., Ipswich, MA). Primers used in this study were purchased from Sigma-Aldrich Co (St. Louis, MO). DNA fragments obtained for cloning were amplified with Phusion High-Fidelity DNA polymerase (New England Biolabs) by using the following touchdown PCR protocol: 1 cycle of 95°C for 5 min, 29 cycles of 95°C for 30 sec, 70°C to 55°C (-5°C/cycle) for 30 sec, 72°C for 2 min, 29 cycles of 95°C for 30 sec, 55°C for 30 sec, 72°C for 30 sec, and 1 cycle of 72°C for 7 min.

### Construction and Complementation of the TMM001 Strain

Matched adaptamers containing 3’ enzyme restriction sites and 5’ complementary sequences were amplified via touchdown PCR. The sequences of the PCR primers were as follows: Δ*tonB* US forward primer (AAG CTA GCC CTC GGC GCG GCG ATC CGC GAC GT) (underlined sequence indicates *Nhe*I site); Δ*tonB* US reverse primer (CGG TAT TGC CGA GAT TAA CGG TGC GGC ACG TCG T); Δ*tonB* DS forward primer (ACG ACG TGC CGC ACC GTT AAT CTC GGC AAT ACC G); and Δ*tonB* DS reverse primer (CCA AGC TTT ACG AGC ATG ACG TCG ACG AGC GGC GTC ATG TTG) (underlined sequence indicates *Hind*III site). The adaptamers were fused together via splicing by overlap extension (SOE) PCR to create a 1794-bp chimeric fragment containing sequences flanking the *tonB* gene plus its first 33 codons. The chimeric fragment was digested with *Nhe*I and *Hind*III and ligated into the pMo130 vector to create the allelic exchange plasmid pTonB-allex. The pTonB-allex plasmid was then transformed into *E*. *coli* S17-1 and introduced into *B*. *mallei* via conjugal transfer. Merodiploids were selected based on their growth on kanamycin (Km) and polymyxin B (Pbx) agar plates and ability to turn yellow after exposure to pyrocathecol. Single deletion mutants were counter selected on YT agar supplemented with 5% sucrose and 200 μM FeSO_4._

After the *tonB* mutant was screened for Pxb resistance and Km sensitivity, deletion was confirmed via PCR amplification, followed by sequencing of the *tonB* gene and flanking DNA regions by using the following primers: confirmation forward primer (5’ GCG CCA CGC GGC CGA TTG CCG CTT TCT) and confirmation reverse primer (ACA GAA CCG TGC CGT CGC TTT). To restore the *tonB* mutant (renamed TMM001) to wild-type function, pMo168 carrying a functional *tonB* gene plus its native promoter was used for complementation. Briefly, a fragment containing the wild-type *tonB* gene plus approximately 120 bp of its upstream sequence flanked by enzyme restriction sites was amplified by using the following PCR primers: complementation forward primer (CCG CTA GCC TGA TTT TCC GCA AGT GAT GCA GCA CT) (underlined sequence indicates *Nhe*I site) and complementation reverse primer (CCA AGC TTT TAA TCG GTC AGA GTG AAG TCA TAA GGC) (underlined sequence indicates *Hind*III site). The fragment was then digested with *Nhe*I and *Hind*III and ligated into the pMo168 plasmid to create pTonB-comp. After transformation into *E*. *coli* S17-1, pTonB-comp was introduced into *B*. *mallei* via conjugal transfer. TMM001 containing pTonB-comp was isolated via selection on LBG + Km agar plates and confirmed by PCR amplification, followed by sequencing of the region flanking the *tonB* gene by the same primers used to confirm the *tonB* mutation.

### Growth Kinetics Assay

Overnight cultures were used to inoculate 50 mL of LBG with 6 x 10^6^ CFU of each strain. Inoculated cultures were then incubated with agitation at 37°C. At the indicated time points, 1 mL aliquots from each culture were taken to measure optical density at 600 nm. Individual data points represent the OD_600_ mean ± standard deviation (SD) of three independent experiments. A significant difference due to treatment over time was ascertained via two-way ANOVA. Significant differences (p ≤ 0.05) of each OD_600_ reading was determined at every time point compared to wild type using one-way ANOVA followed by Dunnett’s multiple comparison test.

### Iron Utilization Assay

Overnight cultures were diluted to 1 x 10^5^ CFU/ml in LBG + 200 μM of 2,2’-dipyridyl and poured onto plates, as previously described [23]. Disks containing iron sources were placed on the surface of the LBG plates, which were incubated at 37°C for 48 h. Disks contained 10 μL of the following compounds at the specified concentrations: hemin, 8.0 μM; hemoglobin, 4.5 μM; myoglobin, 4.5 μM; transferrin, and lactoferrin, both at 30 μM or FeSO_4_, 10 mM [23]. Iron utilization was quantified by measuring the diameter of growth around the disk.

### Siderophore Secretion Assay

Ten μl samples of overnight cultures, grown in LBG or LBG + 200 μM FeSO_4_ or 200 μM 2,2’-dipyridyl, were spotted onto CAS agar plates and incubated at 37°C. Halos were then monitored and the diameter of color change was measured over the course of the next 4 days. For the CAS agar, solutions were prepared as previously described [24]. An unpaired *t* test with equal standard deviation was performed on halo measurements to ascertain a significant difference (p ≤ 0.05) between the strain-specific halos produced.

### Animal Studies

Female, 6- to 8-week-old BALB/c mice obtained from Harlan Laboratories (Indianapolis, IN, USA) were housed in microisolator cages under pathogen-free conditions. Animals were provided with rodent feed and water *ad libitum* and maintained on a 12 h light cycle. Before experiments, mice were afforded an adaption period of at least 1 week. Humane endpoints were strictly observed and time of death was recorded upon death of the animal or at the study’s end. Animals were observed closely throughout the study for clinical symptoms (immobility, dyspnea, paralysis) and moribund animals were anesthetized and then euthanized via cervical dislocation.

### Survival Study

Anesthetized BALB/c mice (n = 8 per treatment) were inoculated i.n. with the indicated CFU of TMM001, grown in LBG ± 200 μM FeSO_4_ and diluted in phosphate-buffered saline (PBS) in a total volume of 50 μL (25 μL/ naris). Mice were monitored and deaths recorded over a period of 14 days. Survival curves were generated and analyzed by using the Kaplan-Meier method. A significant difference (p ≤ 0.05) in survival curves was ascertained via a log-rank test.

### Colonization Study

Anesthetized mice (n = 8 per treatment) were challenged i.n. with 1.5 x 10^4^ CFU/50 μL of the *B*. *mallei* bioluminescent reporter strain CSM001, and TMM001 in LBG ± 200 μM FeSO_4_. At 24, 48 and 72 h post challenge, BALB/c mice were euthanized and necropsied for organ collection. The lungs, liver and spleen were homogenized in 1 mL of PBS by using a tissue grinder (Covidien, Mansfield, MA), and then the bacteria were enumerated by standard plate counts on LBG + 200 μM FeSO_4_. Significant differences (p ≤ 0.05) in colonization at 24 and 48 h were individually determined via one-way ANOVA followed by Tukey’s multiple comparisons test. Significant difference (p ≤ 0.05) in colonization at 72 h was extrapolated by using an unpaired *t* test with equal standard deviation.

### Vaccine and Cross Protection Study

Anesthetized mice (n = 8 per treatment) were immunized i.n. with PBS or the indicated CFU of TMM001 diluted in PBS in a total volume of 50 μL (25 μL/ naris). Mice were challenged 21 days post immunization with 1.5 x 10^5^ CFU (~220 LD_50_) of CSM001 (LD_50_ of 6.81x10^2^ CFU) or 9 x 10^2^ CFU (~3 LD_50_) of wild-type *B*. *pseudomallei* K96243 (LD_50_ of 3.12x10^2^ CFU) [25,26], diluted in PBS in 50 μL (25 μL/ naris). Mice were monitored, and deaths were recorded until the end of the study. Survival curves were generated and analyzed by the Kaplan-Meier method. A significant difference (p ≤ 0.05) in survival curves was ascertained via log-rank test. To find significant differences in individual treatment, when compared to the PBS-treatment control, an additional log rank test was employed in which an adjusted definition of significance (p ≤ 0.05/ the number of pair wise comparisons) was used.

### *In Vivo* Imaging Evaluation

Bioluminescent images were acquired on an IVIS Spectrum (Caliper Corp., Alameda, CA, USA), as previously described [25]. Briefly, anesthetized BALB/c mice placed in the isolation chamber were transferred to the imaging chamber, which was then connected to an internal anesthesia delivery system that maintained 1–2% isoflurane. Bioluminescence signaling was measured after three minutes’ exposure with no excitation (filters blocked) and an open emission filter to capture all luminescent signals from labeled bacteria. To depict the differences in intensity of the signal, bioluminescence was represented in the images with a pseudo-color scale ranging from red (most intense) to violet (least intense). Scales were manually set to the same values for every comparable image to normalize the intensity of the bioluminescence across time points.

### *B*. *mallei-*Specific Immunoglobulin Analysis

Serum extracted from PBS or TMM001-vaccinated BALB/c mice at 21 days post treatment, was evaluated for *B*. *mallei-*specific IgG1, IgG2a and IgM using the Ready-Set-Go! ELISA Kit (Affymetrix eBioscience, San Diego, CA) as instructed by the manufacturer. Briefly, microplates (Costar, Cambridge, MA) were coated with 10 μg/ml of heat inactivated *B*. *mallei* and incubated overnight at 4°C. Wells were then washed twice with PBS, 0.05% Tween-20, and then blocked over night with the Assay Buffer provided in the kit. After the wells were washed, a 1:10,000-fold dilution of sera samples was added to the appropriate wells, followed by the detection antibody provided by the kit. After 3 h incubation, the wells were washed four times before 100 μL of the substrate solution was added. After 15 min incubation, 100 μL of stop solution consisting of 2 N H_2_SO_4_ was added, and absorbance was measured at 450 nm with the Epoch microplate spectrophotometer (Winooski, VT). An unpaired Student’s *t* test was performed to ascertain a significant difference (p ≤ 0.05) in *B*. *mallei*-specific Ig levels between the PBS and TMM001*-*treated mice.

### Histopathological Evaluation

At the indicated time points, necropsies were performed to collect the lungs, liver and spleen. Organs were instilled with 10% formalin, paraffin-embedded, and processed for histopathology. Hematoxylin and eosin-stained slides were examined and blindly scored by a pathologist for the follow observations: perivascular and peribronchial inflammatory infiltrates, necrosis and microabscesses in the lung; granulomas, necrosis and histocytosis in the spleen; and inflammation and necrosis in the liver. Severity of pathology was scored using the following combined scale: 0 (unremarkable), 1 (minimal), 2 (mild), 3 (moderate) and 4 (severe). Pathology scores were combined with a percent factor associated with the extent of the damage (0–25%, 25–50%, 50–75%, 75–100%) and added together to give the total score for each organ. Each image is representative of three replicates per treatment. A two-way ANOVA was performed on each organ individually to assess a significant difference in treatment over time. Student’s *t* test was performed to ascertain a significant difference (p ≤ 0.05) between the treatments of each organ, individually, at 0 and 48 h.

### Cytokine Quantification

At the indicated time points following challenge, whole blood was collected by cardiac puncture. The blood was stored in microvette tubes without anti-coagulant and incubated at room temperature for 20 min to permit clotting. Serum was collected after centrifugation of the tubes and stored at -80°C. Samples were inactivated as previously described [27] and verified for sterility. Serum chemokine/cytokine levels were measured by using the murine bioplex ELISA kit (BioRad Bio-Plex Pro Mouse Cytokine 23-plex Assay) according to the manufacturer’s specification. Serum sample were diluted 1:4 in PBS and expression of the following molecules was determined: interleukin (IL)-1α, IL-1β, IL-2, IL-3, IL-4, IL-5, IL-6, IL-9, IL-10, IL-12 (p40), IL-12 (p70), IL-13, IL-17A, eotaxin, granulocyte–colony-stimulating factor (G-CSF), granulocyte–macrophage colony-stimulating factor (GM-CSF), gamma interferon (IFN-γ), keratinocyte-derived chemokine (KC), monocyte-chemotactic protein (MCP-1), macrophage inflammatory protein (MIP)-1α, MIP-1β, RANTES, and (tumor necrosis factor) TNF-α. Data values represent the mean ± the SEM of three animals per treatment and were ascertained as previously described [27]. Out of range values above the asymptote of equation (> OOR) we set to the highest extrapolated value to provide a conservative estimate that allowed statistical analysis. A significant difference (p ≤ 0.05) in individual serum cytokine levels in PBS vs. TMM001*-*treated mice was determined by using the Mann-Whitney test.

## Results

### Mutant Construction and Initial Characterization

A previously described method for genetic manipulation via allelic exchange was used to create an unmarked *tonB* mutant in the *B*. *mallei* strain ATCC 23344 [22]. To ensure the mutant phenotype was not due to polar effects incurred during mutagenesis, the TMM001 was transformed with the plasmid pTonB-comp, which carries the intact *tonB* gene plus its native promoter (Table 1). Unlike the wild type, TMM001 appears as bright yellow colonies that discolor the surrounding media when grown in Luria Bertani + 4% glycerol (LBG) plates (S1 Fig). This phenotype in iron transport mutants has been attributed to the unregulated production and accumulation of iron-bound siderophores, which are yellow-to-brown in color, in contrast to uncolored iron-free siderophores [28,29,30]. The wild-type phenotype was restored when TMM001 was complemented, which grew as muted yellow-beige colonies with no media discoloration.

### Growth Rate Analysis

**Fig 1:**
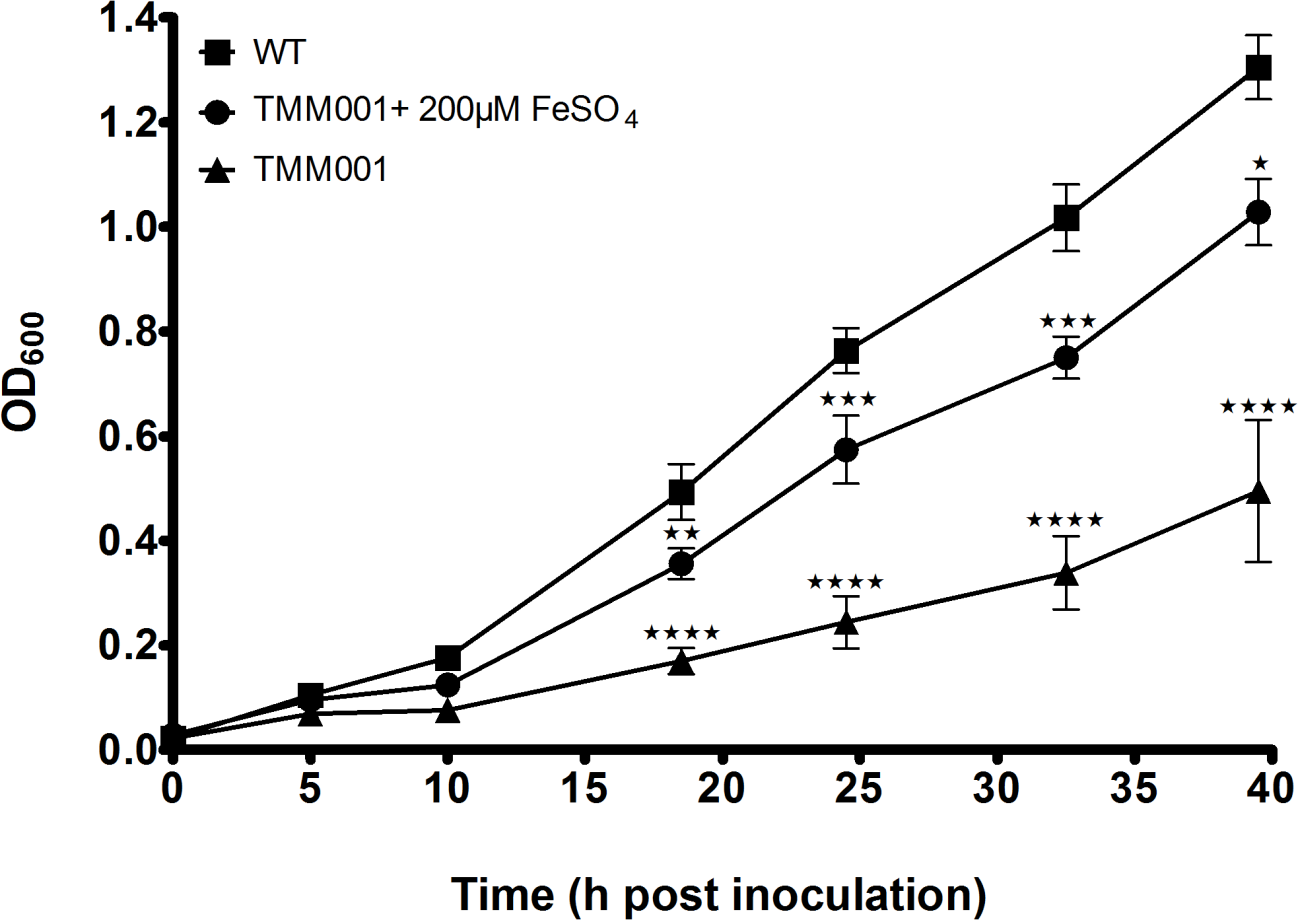
TMM001 attenuated growth kinetics is partially rescued by iron supplementation. Overnight cultures of wild type (solid square) and TMM001 (solid circle) were diluted 1:100 in 50 mL of Luria broth with 4% glycerol (LBG). An additional overnight culture of TMM001 (solid triangle) was diluted 1:100 in 50 mL of LBG + 200 μM FeSO_4_. At the indicated time points, optical densities at 600 nm of all strains were measured. The average with their SD is representative of three independent experiments. Statistical significance was determined by Dunnett’s test of multiple comparisons relative to wild-type. ^★★^ p ≤ 0.001, ^★★★^ p ≤ 0.0001, ^★★★★^ p ≤ 0.0001.

To determine the effect of the *tonB* deletion on growth rate and iron requirement, growth curves were performed with the following strains and broth conditions: wild type in LBG, TMM001 in LBG ± 200 μM FeSO_4_ (Fig 1). When grown in LBG, TMM001 exhibited a reduced growth rate, displaying a longer lag phase, compared to that of the wild type. When grown in LBG + 200 μM FeSO_4_, the growth rate of the TMM001 increased substantially approaching that of the wild type. Notably, TMM001 grown in iron-supplemented media maintained wild-type growth rates showing statistically significant differences only after 25h of growth.

### Siderophore Secretion Assay

To determine if the deletion of *tonB* in *B*. *mallei* resulted in differential siderophore production, both the wild-type and TMM001 were seeded on CAS agar. The CAS media was used because when strong iron chelators, such as siderophores, are secreted, they are able to strip the dye complex of iron, which results in the formation from blue to orange/yellow zones (S2 Fig). Siderophore secretion zones were measured after 96 h and calculated as the diameter of the halo minus the diameter of bacterial colony on the filter disk. TMM001 produced significantly larger halos (33.3 ± 0.5 mm) compared to those of the wild type (12.3 ± 0.6 mm). These results are in line with previously studies that show iron transport mutants hypersecrete siderophores in a futile attempt to acquire iron [28,30,31,32,33,34,35].

### Iron Utilization Assay

A disk diffusion assay was performed to examine the ability of the TMM001 to utilize the following sources of iron: FeSO_4_, hemoglobin, hemin, lactoferrin, and transferrin. Iron assimilation was determined by measuring the diameter (mm) of bacterial growth around the disk containing specific iron sources placed on iron-depleted media (S1 Table). The wild-type strain was able to grow by utilizing all iron sources, while TMM001 was only capable of utilizing FeSO_4_, the only iron source acquired by a TonB-independent process.

### *In Vivo* Survival Study

**Fig 2:**
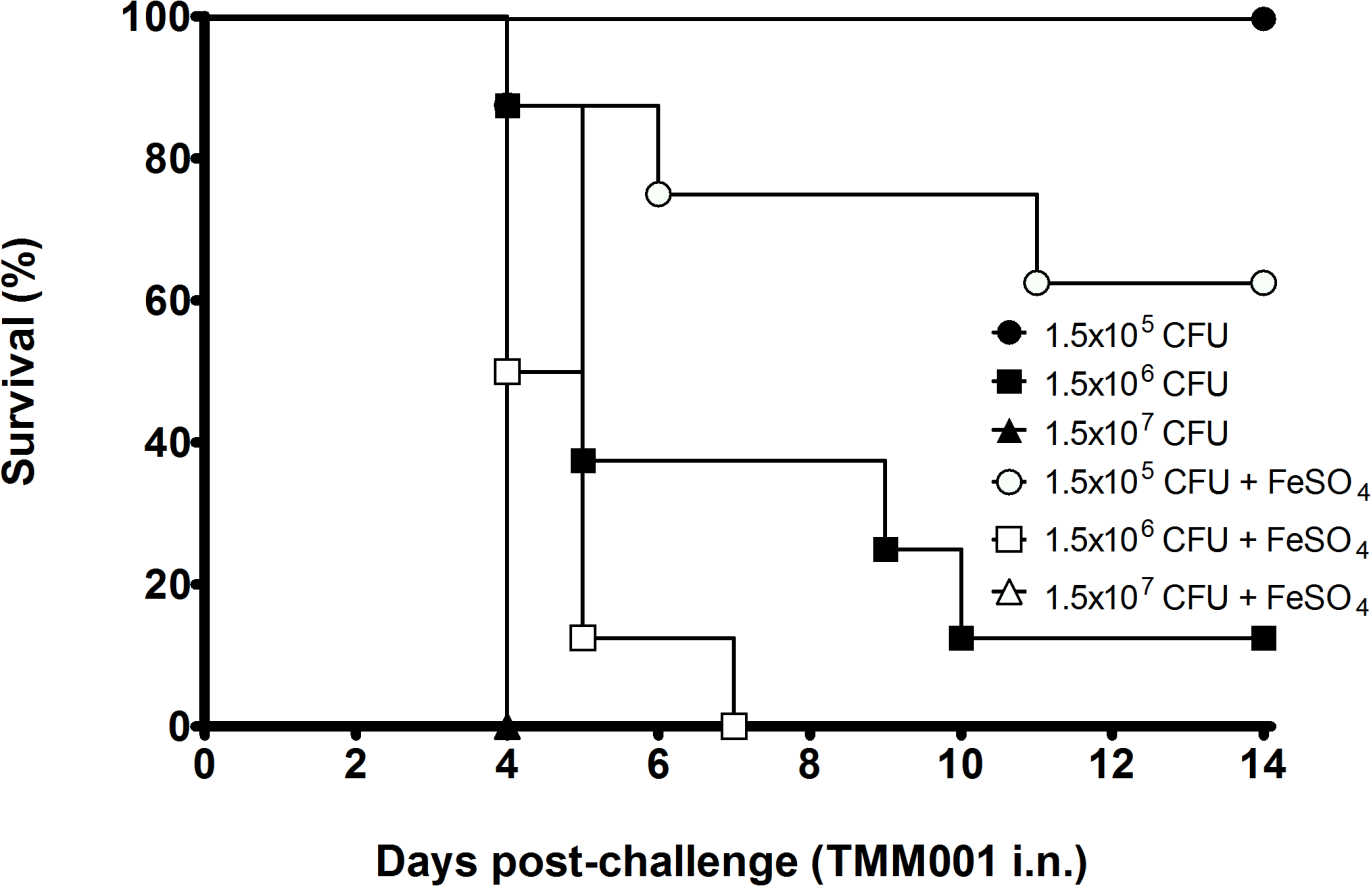
Attenuated virulence of TMM001 is partially rescued by iron supplementation. Mice (n = 8) were challenged i.n. with 1.5 x 10^5^ CFU (solid circle/open circle), 1.5 x 10^6^ CFU (solid square/open square) or 1.5 x 10^7^ CFU (solid triangle/open triangle) of TMM001 grown in LBG with (open) or without (closed) 200 μM FeSO_4_. The statistical significance of differences in survival times was determined by plotting Kaplan-Meier curves, followed by a log rank test. ^★★★★^ p ≤ 0.0001.

In previous characterization studies of our acute respiratory murine inhalational glanders model, we observed that the 50% lethal dose using *B*. *mallei* strain ATCC 23344 was 7.4 x 10^4^ CFU/50 μL (Torres lab experimental data). To establish the role of *tonB* in *B*. *mallei* virulence, we challenged BALB/c mice intranasally (i.n.) with 1.5 x 10^5^ CFU, 1.5 x 10^6^ CFU and 1.5 x 10^7^ CFU of TMM001 grown in LBG ± 200 μM FeSO_4_ and monitored them for survival up to day 14. The Kaplan-Meier curve shows an inverse correlation between the dose and/or iron concentration and the mouse survival rate (Fig 2). Despite growth conditions, all BALB/c mice challenged with 1.5 x 10^7^ CFU of TMM001 succumbed to infection by 4 days post challenge. At lower doses, the effect of supplementing TMM001 with 200 μM FeSO_4_ on survival was still apparent. At day 14, survival increased from 62.5% to 100% and 0% to 12.5% when BALB/c mice received a challenge dose of 1.5 x 10^5^ CFU and 1.5 x 10^6^ CFU of the TMM001, respectively, which was grown in LBG alone.

### Colonization Study

**Fig 3:**
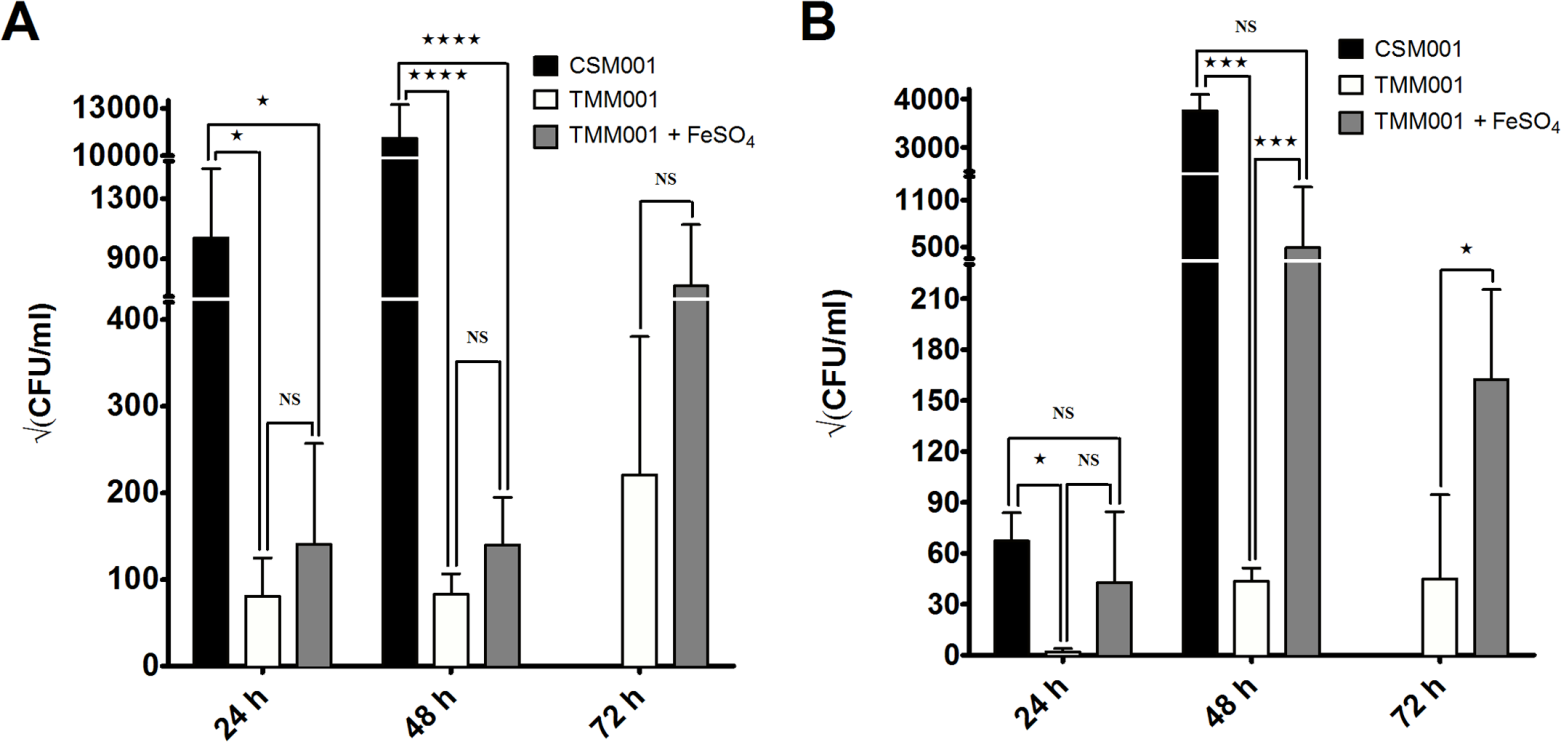
Colonization of target organs by TMM001 is partially rescued by iron supplementation. Bacterial burden in the lungs (A) and spleen (B) of mice infected with CSM001 and TMM001 grown ± 200 μM FeSO_4_ at 24, 48 and 72 h post infection. Bars plotted with their SD represent the mean of three independent experiments. Significant differences in colonization at 24 and 48 h were individually ascertained via one-way ANOVA followed by Tukey’s multiple comparisons test. Significant difference in colonization at 72 h was extrapolated by using an unpaired *t* test with equal SD. ^★^ p ≤ 0.05, ^★★★^p ≤ 0.001, ^★★★★^p ≤ 0.0001, ns = no significance.

We next enumerated bacterial counts in infected organs to determine the role of TonB in *B*. *mallei’s* ability to disseminate and colonize target tissues. BALB/c mice challenged i.n. with 1.5 x 10^4^ CFU of the wild-type CSM001 or TMM011 grown in LBG ± 200 μM FeSO_4_ were euthanized at 24, 48 and 72 h post challenge. At each time point, the lungs and spleen were processed and plated for CFU quantification. Compared to CSM001, the numbers of TMM001 recovered from the lungs were significantly reduced at 24 h (^★^ p ≤ .05) and 48 h (^★★★★^ p ≤ .0001), independent of growth conditions (Fig 3A). A similar trend was observed in the spleen with significantly reduced numbers of TMM001 compared to the CSM001 at 24 h (^★^ p ≤ .05) and 48 h (^★★★^ p ≤ .001) (Fig 3B). When grown in LBG + 200μM FeSO_4_ prior to challenge, TMM001 resembled CSM001, showing no statistical difference in the number of bacteria recovered from the lungs. However, a statistical difference was seen in the recovery of TMM001 grown in FeSO_4_ in the spleen at 72 h (^★^ p ≤ .05) (Fig 3A and 3B). BALB/c mice challenged with the CSM001 expired before the 72 h time point and data are not presented.

### Protection Studies

To evaluate the protective efficacy of TMM001 against CSM001 challenge, BALB/c mice received PBS, 1.5 x 10^4^ CFU or 1.5 x 10^5^ CFU of TMM001 (grown in LBG only), via the i.n. route. At 21 days post-immunization, vaccinated mice were challenged i.n. with 1.5 x 10^4^ CFU of CSM001. The wild-type homolog CSM001, containing a luminescent reporter, was used to assess the protective potential of TMM001 via real-time *in vivo* monitoring. All infected PBS-treated BALB/c mice died by day 4, presenting with a calculated median survival of 3 days post challenge (S3 Fig). In contrast, infected mice immunized with TMM001 at a dose of 1.5 x 10^5^ CFU or 1.5 x 10^4^ CFU showed 100% (^★★★^ p = 0.0003) and 87.5% (^★★★^ p = 0.0003) survival, respectively.

Dissemination and colonization of CSM001 was monitored in TMM001-treated and naïve BALB/c mice using IVIS at 72 h post challenge and every 7 days thereafter until the experiment ended. At 72 h post challenge, PBS-treated BALB/c mice exhibited a luminescent signal associated with anatomical locations corresponding to the lungs, liver, spleen and brain. However, this signal was not detected at similar locations in BALB/c mice immunized with TMM001 (S4 Fig). To evaluate whether TMM001 immunization resulted in the production of sterile immunity, BALB/c mice surviving the experimental challenge were euthanized and organs harvested to be analyzed for gross pathology and bacterial persistence. Although the lungs and livers showed no signs of evident pathology, BALB/c mice presented with splenomegaly accompanied by multiple splenic abscesses (S5 Fig, panels D-F), which mirrors spleens at stage 3 of murine melioidosis infection, as we previously described [27]. Bacterial counts were only recovered from the spleens of mice immunized with a dose 1.5 x 10^5^ (334,666 ± 70,465 CFU per spleen) and 1.5 x 10^4^ (61,917 ± 18,217 CFU per spleen) CFU of TMM001. Based on the phenotypic yellow pigment of the colonies, polymyxin B resistance and kanamycin sensitivity, we were able to conclude that all bacteria recovered were TMM001 and not CSM001.

**Fig 4:**
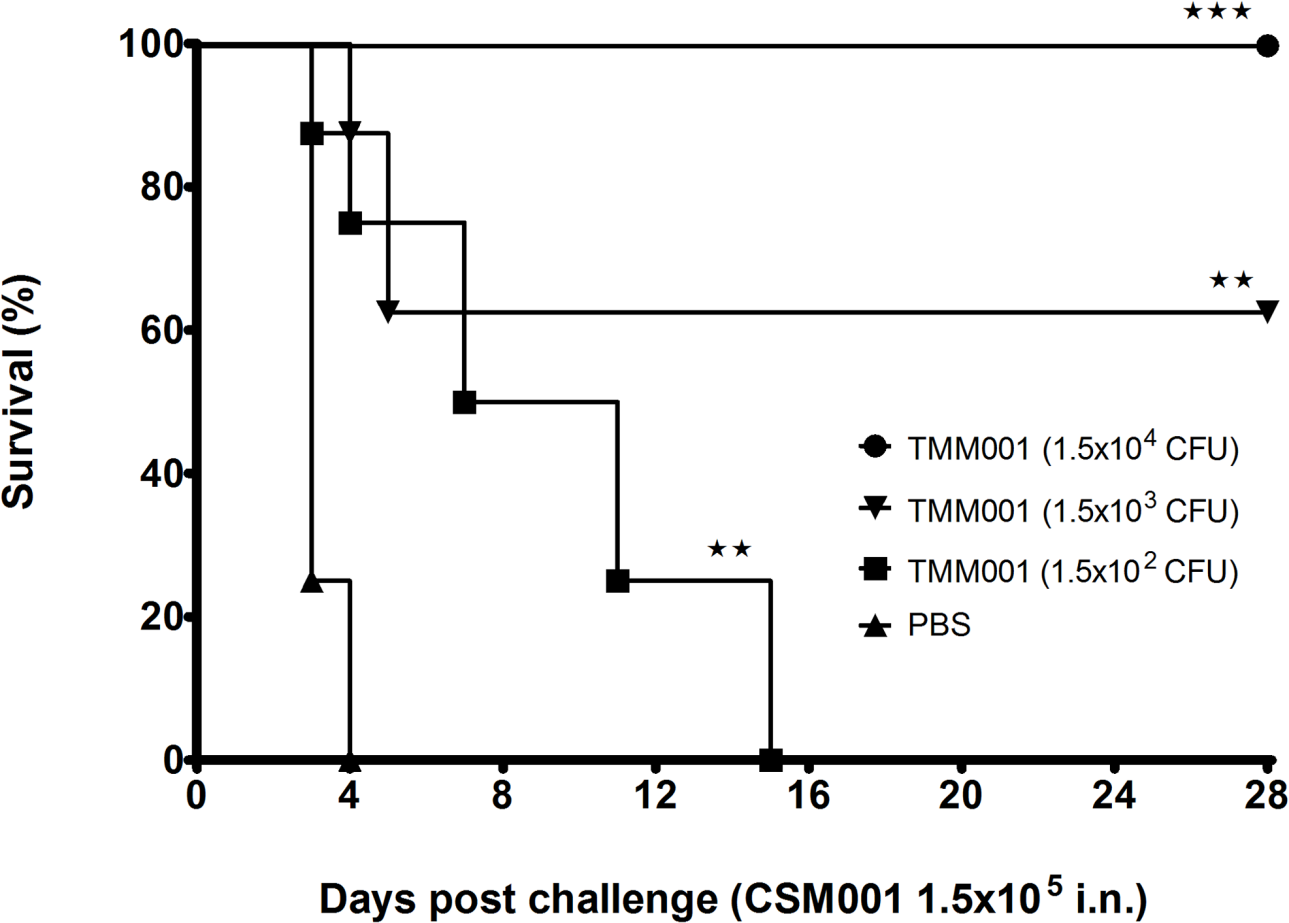
TMM001 (1.5x10^4^ CFU) provides 100% protection against CSM001 challenge. Mice were immunized i.n. with PBS (solid triangle), 1.5 x 10^4^ CFU (solid circle), 1.5 x 10^3^ CFU (solid inverted triangle), or 1.5 x 10^2^ CFU (solid square) of TMM001. Three weeks later, mice were challenged with 1.5 x 10^5^ CFU of CSM001. The statistical significance of differences in survival times was determined by plotting Kaplan-Meier curves, followed by a log rank test. ^★★★^ p ≤ 0.001, ^★★^ p ≤ 0.01.

In an attempt to eliminate persistence of the attenuated TMM001 strain, as well as to reduce organ pathology, an attenuated strain titration study was initiated to identify the lowest immunization dose that still provided 100% protection. The TMM001 titration study used the following CFUs for immunization: 1.5 x 10^4^, 1.5 x 10^3^ and 1.5 x 10^2^. Twenty-one days post-immunization, three mice from each immunization group were euthanized, and organs and serum were harvested for histopathological and cytokine analysis. Forty-eight hours after CSM001 (1.5 x 104 CFU) challenge, an additional 3 mice from each treatment were euthanized, and organs and serum were harvested for histopathological and cytokine analysis. As previously observed, all PBS-treated mice challenged with *B*. *mallei* CSM001 died by day 4, with a median survival of 3 days (Fig 4). The titration curve exhibits a significant dose-dependent increase in survival in TMM001*-*treated mice challenged with CSM001. All mice immunized with 1.5 x 10^2^ CFU expired by day 15, with an increased mean survival of 9 days (^★★^ p = 0.0016). Mice immunized with 1.5 x 10^3^ CFU or 1.5 x 10^4^ CFU, survival up to 28 days increased to 62.5% (^★★^ p = 0.0016) and 100% (^★★★^ p = 0.00016), respectively. Assessment of bacterial burden in surviving animals showed the spleen and, to a lower extent, the liver chronically infected in the TMM001-treated but not the CSM001 strain.

### Development of Humoral Immune Response

**Fig 5:**
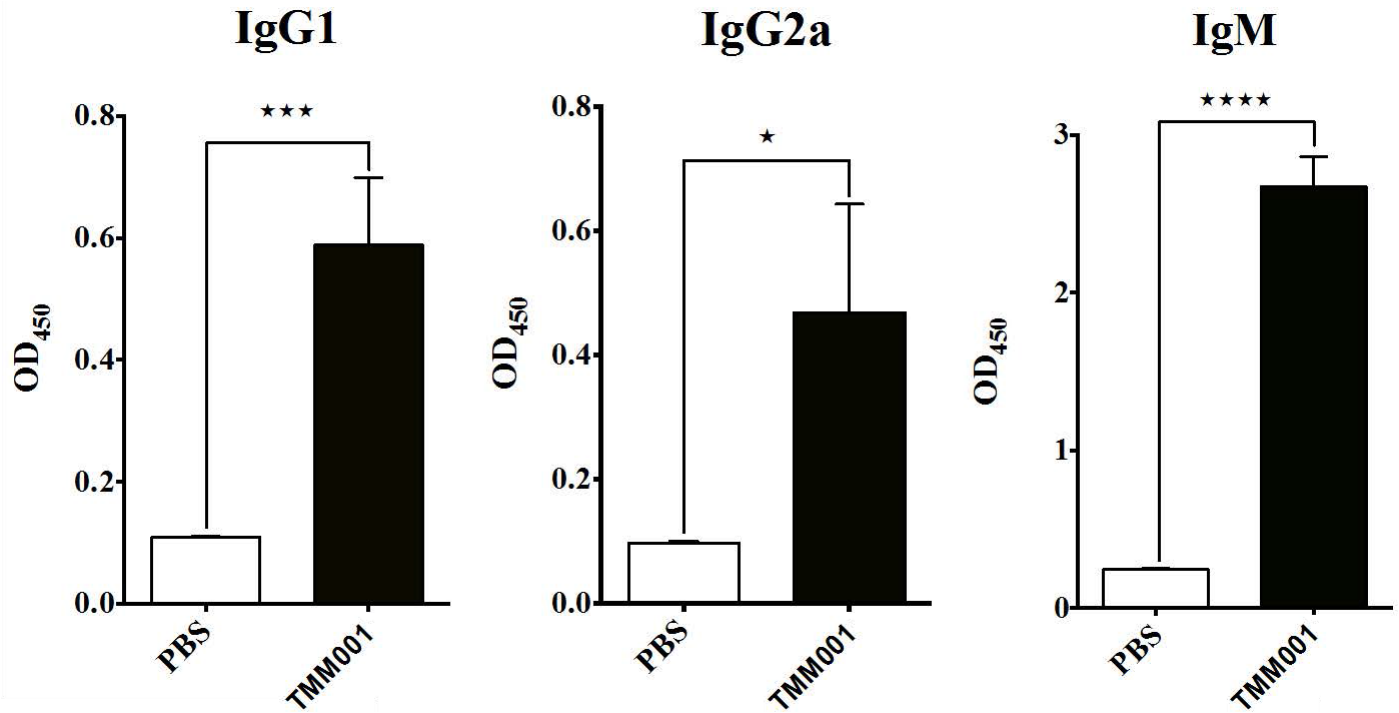
*B*. *mallei-*specific immunoglobulin levels in TMM001- vs PBS-treated mice before challenge. Murine serum samples were taken 21 days post-immunization, diluted 1:10,000 and analyzed for *B*. *mallei-*specific IgG1 (A), IgG2a (B) and IgM (C). Mean ± SEM of three representative animals is plotted. Statistical significance was determined by the unpaired *t* test with equal SD. ^★^ p ≤ 0.05 ^★★★^ p ≤ 0.001, ^★★★★^ p ≤ 0.0001.

The generation of murine humoral immune responses to *B*. *mallei* following treatment with mock (PBS) or TMM001 was determined by analysis of sera using ELISA. Compared to mock-vaccinated mice, sera from TMM001-treated mice had significantly higher titers of *B*. *mallei-*specific IgM and IgG antibodies (Fig 5). Mean differences in absorbance for IgG1, IgG2a, and IgM were 5.4-fold (p = 0.0009), 4.8-fold (p = 0.0106), and 10.9-fold (p = 0.0028) higher, respectively, in TMM001*-*vaccinated mice.

### Histopathological Analysis

**Fig 6:**
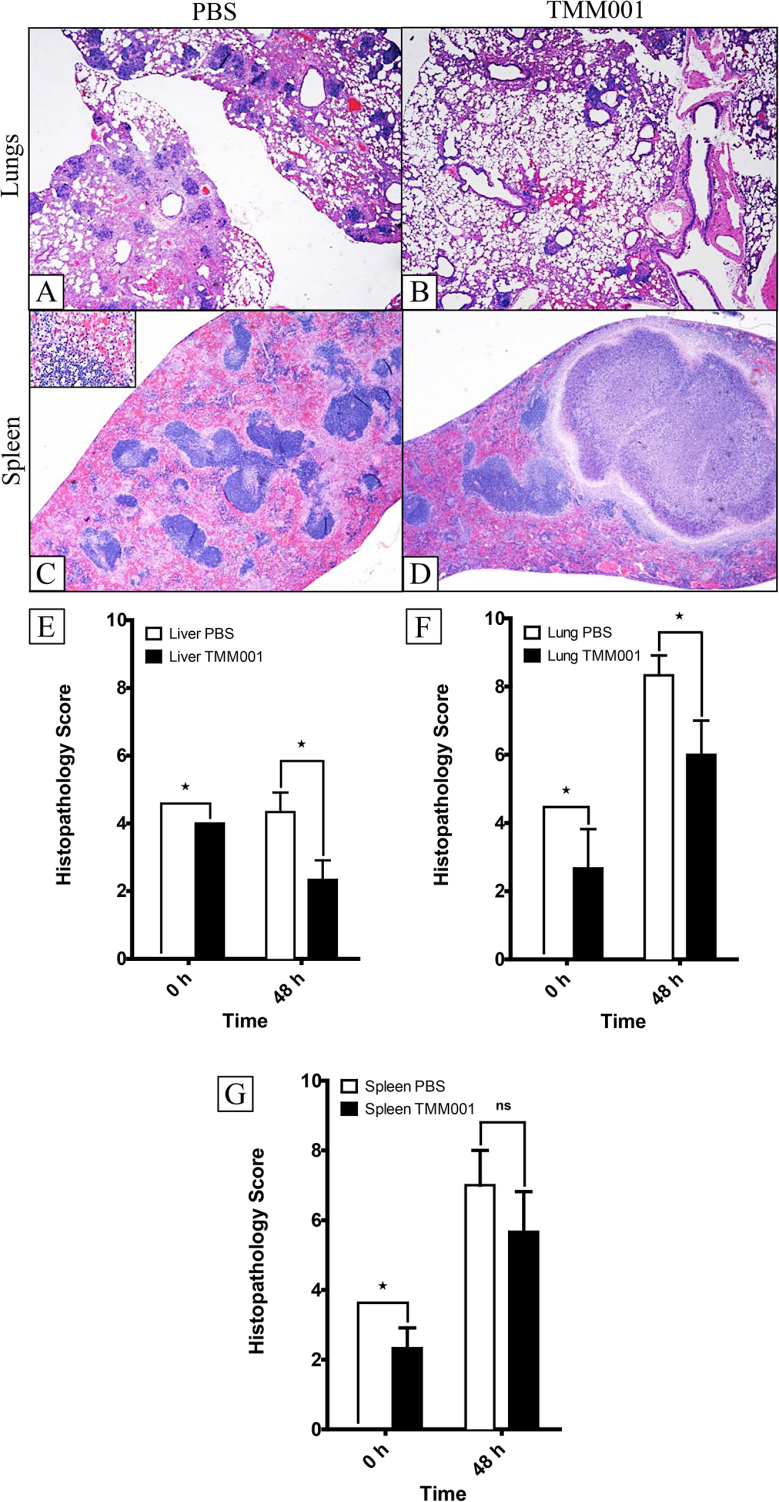
Histopathology of TMM001- vs PBS-treated mice 48 h post challenge. H&E-stained lung (A, B) and spleen (C, D) of CSM001 (1.5 x 10^5^ CFU) challenged mice previously immunized with PBS (A, C) or TMM001 (1.5 x 10^4^ CFU) (B, D). Scale bar = 100μM. Histological scores were assigned for the liver (E), lungs (F), and spleen (G) tissue sections after microscopic examination. Mean ± SEM, representative of three animals, is plotted. Statistical significance was determined by the Mann-Whitney test. ^★^ p ≤ 0.05.

The mouse tissues (lungs, liver and spleen) from the TMM001 titration study (n = 3 per treatment) at 0 h and 48 h post-challenge were processed for histology. Representative images of the lungs, liver and spleen from PBS- and TMM001 (1.5 x 10^4^ CFU)-immunized mice are presented in S6 Fig. At 0 h, the lungs, livers and spleens of PBS-treated mice were unremarkable, presenting as normal healthy tissue with normal architecture (S6 Fig, panels A-C). BALB/c mice immunized with TMM001 presented with mild-to-moderate changes in pathology: perivascular and peribronchial inflammatory infiltrates in the lung sections (S6 Fig, panel D), hepatitis with multifocal necrosis and scattered abscesses in the liver sections (S6 Fig, panel E), and necrosis of follicles and accumulation of neutrophils in spleen sections (S6 Fig, panel F). At 48 h post challenge with CSM001 (1.4 x 10^4^ CFU), PBS-treated mice showed moderate-to-severe pathological changes, such as abscesses and multifocal inflammatory infiltrates in the lungs (S6 Fig, panel G and Fig 6A), areas of hepatocellular necrosis, occasional abscesses with necrotic cores and areas of focal necrosis in the liver (S6 Fig, panel H), and congestion of the red pulp, proliferation of large foamy macrophages (inset of Fig 6, panel C) and necrosis affecting the mantle zone (S6 Fig, panel I and Fig 6, panel C). Similarly, TMM001*-*immunized mice showed moderate-to-severe changes in pathology, but with a few differences. In the lungs, large, multifocal inflammatory infiltrates, as well as abscesses, were present with focal consolidation observed as well (S6 Fig, panel J and Fig 6, panel B). The liver presented with hepatitis and multiple foci of hepatocellular necrosis (S6 Fig, panel K), and large granulomas were formed in the spleen (S6 Fig, panel L and Fig 6, panel D). Histopathology scores showed significant differences due to treatment over time in the lungs (^★★★★^ p ≤ 0.0001), liver (^★★★★^ p ≤ 0.0001) and spleen (^★★^ p ≤ 0.001). When comparing the differences in treatment at 0 h and 48 h, the lungs (^★^ p = 0.05), liver (^★^ p = 0.05) and spleen (^★^ p = 0.05) showed a robust trend toward significance (Fig 6, panels E-G). Overall, TMM001-immunization alone does cause some histopathology as evident by the histopathology at 0 h. That being said, PBS-immunized animals exuded more extensive pathology 48 h after CSM001 challenge compared to TMM001-immunized animals.

### Serum Cytokine Analysis

**Fig 7:**
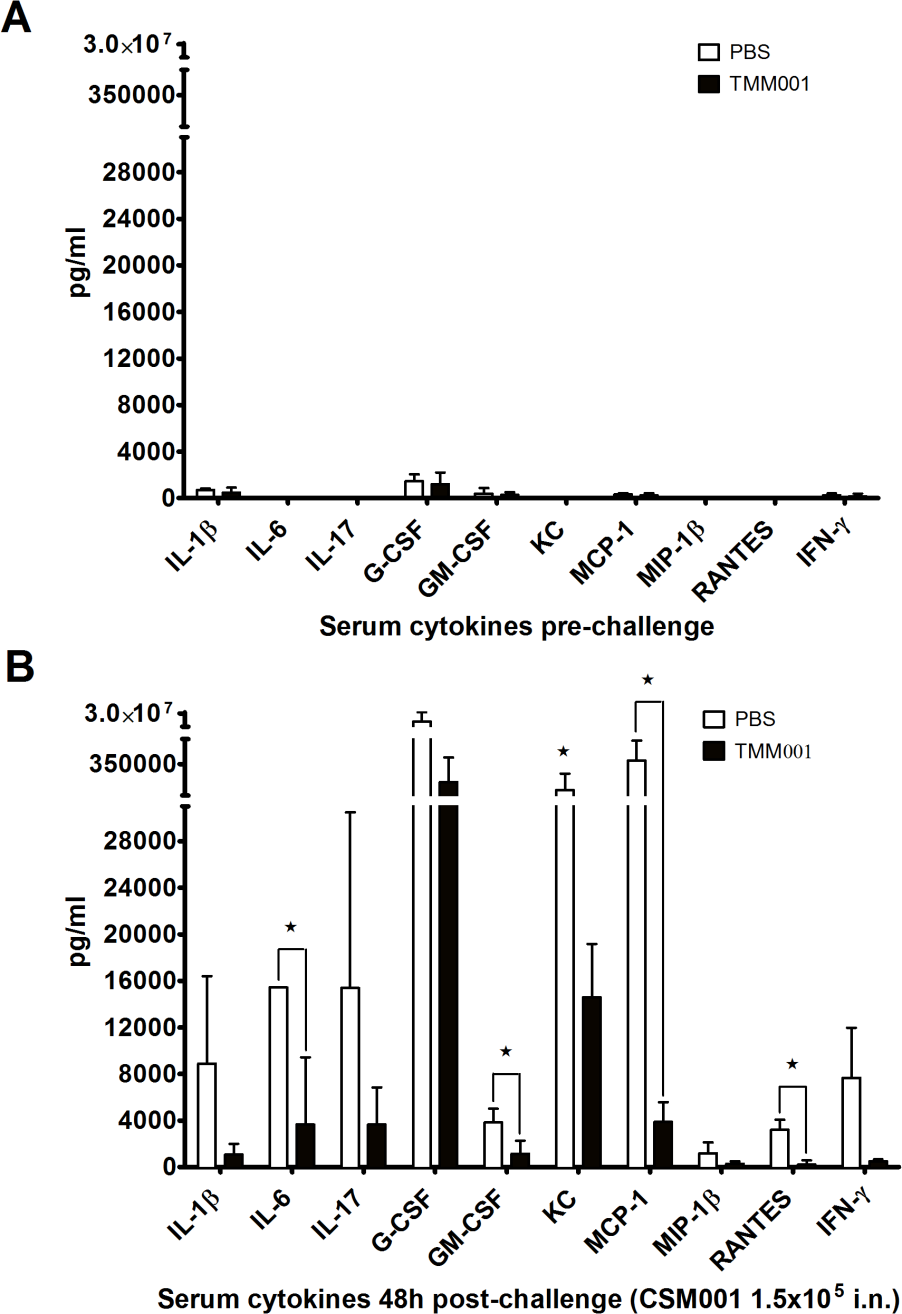
Treatment with TMM001 reduces the pro-inflammatory cytokine and chemokine response to *B*. *mallei* challenge. Serum cytokine/chemokine profile of PBS- or TMM001-treated mice following exposure to *B*. *mallei* CSM001 (1.5x10^5^ CFU) at 0 h (A) and 48 h (B) post challenge. Mean ± SEM plotted are representative of three animals. Statistical significance was determined by one-way ANOVA followed by the Dunnett's test. ^★^ p ≤ 0.05.

Sera that was collected at 48 h post challenge from PBS- and TMM001-treated mice was used to identify pro-inflammatory cytokine and chemokine responses that correspond with disease outcome. Prior to challenge, a similar baseline expression of cytokines and chemokines was detectable in serum of representative animals from both the PBS- and TMM001-immunized animals (Fig 7A). Following CSM001 challenge, the overall cytokine/chemokine expression increased markedly in both PBS- and TMM001-immunized animals (Fig 7B) compared to baseline, consistent with our previous observations of innate immune responses to *Burkholderia* species [27]. An attenuation of the pro-inflammatory serum cytokine/chemokine response to challenge was observed in the TMM001-treated compared to PBS control. The reduction of several pro-inflammatory mediators due to TMM001-treatment was significant, including IL-6 (p = 0.049), GM-CSF (p = 0.037), MCP-1 (p = 0.022), and RANTES (p = 0.032) (Fig 7). A trend for reduction of several other pro-inflammatory IL-1β (*p* = 0.097), G-CSF, (*p* = 0.067) and KC (p = 0.05) due to TMM001-treatment was also observed (Fig 7).

### Cross Protection Study

**Fig 8:**
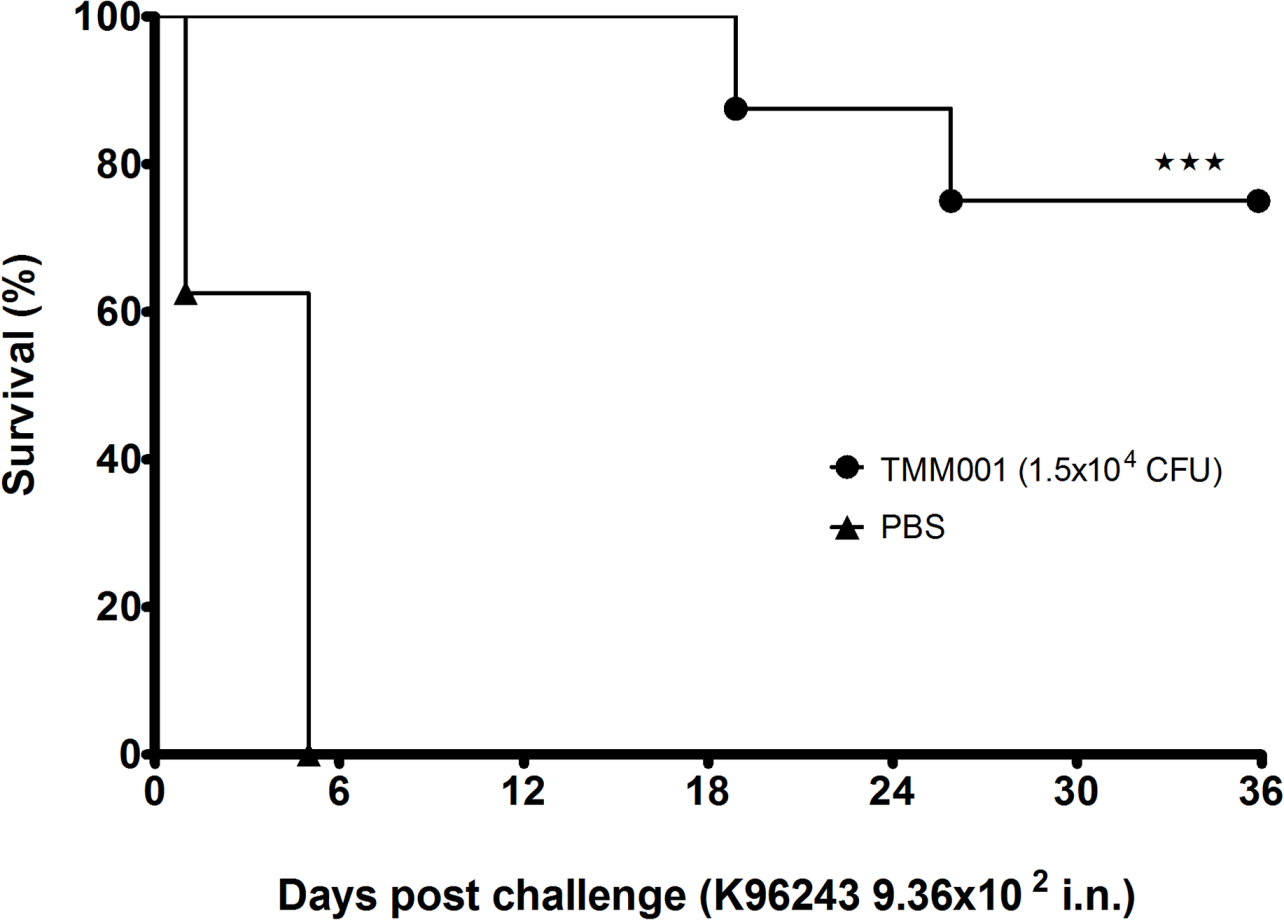
TMM001 provides increased protection against *B*. *pseudomallei* wild-type challenge. Mice were immunized i.n. with PBS (solid triangle) (n = 7) or 1.5 x 10^4^ CFU (solid circle) of TMM001 (n = 8). Three weeks later, BALB/c mice were challenged (day 0) with 9.36 x 10^2^ CFU of *B*. *pseudomallei* K96243. Statistically significant differences in survival times were determined by Kaplan-Meier curves, followed by a log rank test. ^★★★^ p ≤ 0.0001.

We next tested TMM001 for its protective potential against *B*. *pseudomallei* in an acute inhalational model of murine melioidosis. Mice received 1.5 x 10^4^ CFU of TMM001 and at 21 days post-immunization, they were challenged with 9.0 x 10^2^ CFU (3 LD_50_) of *B*. *pseudomallei* strain K96243 [26]. All PBS-treated BALB/c mice died by day 5 post-challenge and displayed a median survival of 5 days (Fig 8). In mice immunized with TMM001, survival was increased to 75% (^★★★^, *p* ≤ 0.001) at the end point of 36 days. As with the previous *B*. *mallei* study described above, the TMM001 strain, but not the wild-type *B*. *pseudomallei*, were recovered from immunized mice who presented with splenomegaly accompanied by abscesses.

## Discussion

To date, immune correlates of protection for *B*. *mallei* and *B*. *pseudomallei* are not clearly defined. Due to their intracellular lifestyle, these pathogens use an array of virulence factors to invade, replicate, and cause pathogenesis from within host cells, which can impede immune detection and, in some cases, protection. An extensive review of the literature suggested to us that an ideal vaccine for both pathogens would induce robust humoral and cell-mediated responses [1,36]. Thus, we decided to examine live attenuated vaccines, as these are often cited as the most efficacious approach to vaccine development against intracellular pathogens because they mimic natural infection, inducing both humoral and cell-mediated immunity, without causing disease. Moreover, exposure to the live attenuated strain allows the immune system to customize a protective response, in addition to generating an immune memory for lifelong protection against infection.

In growth curve experiments, it was found that TMM001 was unable to maintain wild-type growth kinetics (Fig 1). Upon supplementing the culture with free iron, TMM001 exhibited increased growth rates more reminiscent of the wild-type, which is illustrated by a shorter lag phase and prolonged maintenance of wild-type growth kinetics. In a separate growth curve study, full rescue of the wild type phenotype in TMM011 was achieved after both the starter and sub-culture were supplemented with free iron. The correlation between free iron concentration and the growth rate of TMM001 illustrates the importance of TonB as a facilitator of iron transport, which has a direct impact on bacterial fitness.

The results of our survival study show an inverse correlation not only between TMM001 dose and survival but also between concentration of free iron and survival (Fig 2). Compared to the wild-type *B*. *mallei* strain (LD_50_ of 7.5 x 10^4^ CFU), TMM001 is approximately 3-fold more attenuated when grown with FeSO_4_ (LD_50_ of 2.38 x 10^5^ CFU); and when grown in LBG alone, the *tonB* mutant is attenuated by approximately 7-fold (5.59 x 10^5^ CFU). Differences in virulence are consistent with the data of the dissemination study which showed the lowest burden in animals infected with TMM001 and higher bacterial burdens in animals infected with TMM001 grown with FeSO_4_ (Fig 3). In both experiments, FeSO_4_ supplementation failed to fully reverse attenuation of TMM001 *in vivo*. This outcome was not unexpected as the concentration of free iron in the host (10^-24^M [37]) is well below that is needed to sustain bacterial replication. While FeSO_4_ supplementation would prolong its survival and therefore increase its virulence, TMM001 would be unable to sustain the wild-type phenotype once its internal stores of iron are exhausted. Lack of full complementation by FeSO_4_ supplementation can also be attributed to the role of TonB in the import of other substrates. While the majority of TonB-dependent transport systems function to uptake iron, vitamin B_12_, nickel chelates, and carbohydrates can also be transported by this mechanism. Overall, decreased mortality observed in animals challenged with the TMM001 grown in LBG alone illustrates the importance of iron and its TonB-mediated acquisition to virulence.

In a series of TMM001 titration studies, it was empirically determined that a dose of 1.5 x 10^4^ CFU of TMM001 resulted in 100% protection (Fig 4) and CSM001 clearance following challenge. Protected animals developed strong *B*. *mallei*-specific IgG1, IgG2a, and IgM responses (Fig 5), which we attribute to TMM001-mediated protection. The observation and correlation of strong IgG and IgM elicitation and protection are cited often in *Burkholderia* vaccine studies [38,39,40,41]. In human cases, it was found that patients with less severe, localized infection produced detectable *Burkholderia-*specific IgM antibody titers, whereas none were detected in patients suffering from acute disseminated infection [39]. Thus, it is plausible to suggest TMM001 treatment protects against lethal infection by neutralizing bacteria and/or preventing their dissemination to target organs via antibody-mediated mechanisms.

TMM001 immunization resulted in pathological differences that may explain increased survival and protection. In general, histopathological scoring shows a robust trend toward significant differences in the pathology seen in the lungs, liver and spleen of PBS- vs. TMM001*-*immunized animals (Fig 6, panels E-G). Further analysis of these tissues revealed two discriminatory elements of pathologic damage between vaccine treatments. First, despite the finding that the lungs and livers from both PBS- and TMM001-immunized animals displayed some degree of tissue damage, the pathological changes in TMM001*-*immunized mice were much less severe (S6 Fig, panel A). Second, the differential alteration in spleen architecture implied that the PBS- and TMM001-immunized animals responded differently to infection. For example, splenic tissues from PBS-treated mice show a diffuse response to injury (i.e. diffuse severe histiocytosis), while splenic tissue from TMM001*-*immunized mice showed a focal response to injury (i.e. granuloma formation) (S6 Fig, panels C-D). These histological observations suggested to us that immunization with TMM001 may result in the induction of an immune response that produces a different type of tissue damage, in addition to confining infection to prevent disseminated disease, an important cause of morbidity and mortality in many diseases [39,42,43,44].

The histopathological differences between PBS- and TMM001-vaccinated mice suggest that TMM001 reduces disease by attenuating immune-mediate pathology at sites of bacterial proliferation. Our observation that pro-inflammatory cytokine/chemokine responses are attenuated following TMM001 treatment further supports this conclusion. In models of murine melioidosis, it has been established that increased expression of IL-1β and IL-6 follow *B*. *pseudomallei* dissemination and coincide with acute sepsis and mortality [45,46]. Clinical evidence further suggests a correlation between elevated serum levels of IL-1β and IL-6 and poor prognosis in patients with septic melioidosis [47,48,49]. Our previous studies demonstrated that pre-treatment with CpG oligonucleotides protected mice from *B*. *pseudomallei* exposure and reduced pro-inflammatory cytokine/chemokine (e.g. IL-1β, IL-6, G-CSF, KC, MCP-1) expression in the lung [35]. In the study by Judy, *et al*., a moderate pro-inflammatory response was associated with protection while excessive inflammation caused pulmonary pathology [26]. Further, the protective effects of CpG treatment to reduce lung pathology were attributed to a reduction in neutrophil and inflammatory monocyte recruitment [26,50]. Similarly, we have previously shown that the virulence of *B*. *pseudomallei* strains in direct comparisons corresponds with excessive production of pro-inflammatory cytokines and chemokines that recruit neutrophils and monocytes [27]. These observations in clinical and animal model studies support a role for exacerbated pro-inflammatory responses to mediate lung pathology in disease due to *Burkholderia* species. Thus, treatment with TMM001 activates a moderate pro-inflammatory cytokine/chemokine response associated with protective immune responses and attenuates the exacerbation of this response that is associated with neutrophil infiltration and immune-mediated tissue damage.

Lastly, since *B*. *mallei* and *B*. *pseudomallei* are genetically closely related, TMM001 was further tested for its potential to provide protection in an acute inhalational model of murine melioidosis. The significant cross protection seen in TMM001-treated mice provides an optimistic outlook for the development for a single vaccine for both pathogens. Immunization with TMM001 resulted in full protection and clearance of CSM001 when tested in an acute respiratory model of murine glanders. This live attenuated strain is unique not only because it provided full protection against both acute and chronic stages of infection but also because it imparted significant cross protection against *B*. *pseudomallei* infection.

It is hypothesized that the persistence of viable bacteria is key for protective potential. In previous vaccination studies, the failure to provide successful long-term protection has often be attributed to the quick removal of live attenuated strains from the host [51,52]. Thus, we believe the resulting long-term protection is link to the ability of TMM001 to evade rapid clearance. This notion is supported by vaccination studies which reported long-term survivors to be generally colonized at the end of the study [51,52,53]. It is plausible to propose that this persistence increases the accessibility of the immune system to protective antigens or it might contribute to the development of an environment adverse to wild-type colonization via chronic elicitation of the immune response.

Although its persistence is important for its protective potential, TMM001 is able to colonize and maintain in the host. Before securing approval from the division of select agents and toxin (DSAT) for removal of this strain from the Health and Human Services select agent list and becoming a legitimate vaccine candidate, the ability of TMM001 to cause chronic infection needs to be addressed. Studies are now focused on using TMM001 as a backbone to generate a further attenuated strain with the introduction of additional mutations. As TMM001 is only 7-fold less virulent than the wild-type *B*. *mallei* strain, we believe this to be the best strategy for optimization and do not anticipate problems with over-attenuation. Currently we are targeting genes that are contributing to persistence with the intention of developing a more attenuated strain that can persist long enough to elicit a protective immune response without establishing chronic infection. For example, we are focused on genes involved in TonB-independent mechanisms of iron assimilation. Bacterial transport systems that are shown to transport iron in this manner include the following: FbpABC transport system of *Neisseria gonorroeae*¸ SfuABC transport system of *Serratia marcescens*, VctPDGC ABC cytoplasmic membrane transport system of *Vibrio cholera*, etc [Ref]. Looking for homologs of these systems in *B*. *mallei* could provide optimal targets for further attenuation. Overall, we believe the present study represents a significant advancement in the battle against pathogenic *Burkholderia* infections, in which TMM001 could be further optimized to become an effective vaccine against glanders, melioidosis, or other *Burkholderia* infections.

## Supporting Information

S1 Fig TMM001 displays a differential phenotype. Wild-type (lower left), TMM001 (top) and the complemented Δ*tonB* (pTonB-comp) (lower right) were grown on LBG + 200 μM FeSO_4_ for 3 days at 37°C. The figure shows the differences in colony color and modification to the agar media as result of secretion of a pigment by TMM001.
(TIF)

S2 Fig TMM001 displays hyper-secretion of siderophores. Ten microliters of wild-type (A) and TMM001 (B) overnight cultures grown in LBG were spotted on a filter disk placed on CAS agar media. CAS agar plates were then incubated for at 37°C for 96 h.
(TIF)

S3 Fig TMM001 (1.5 x 10^5^ CFU) provides 100% protection against wild-type challenge. Mice (n = 8) were immunized i.n. with PBS (solid triangle), 1.5 x 10^4^ CFU (solid circle) or 1.5 x 10^5^ CFU (solid diamond) of TMM001. Three weeks later, BALB/c mice were challenged with 1.5 x 10^5^ CFU of a *B*. *mallei* reporter strain CSM001. The statistical significance in the different survival times was determined by plotting Kaplan-Meier curves, followed by a log rank test. ^★★★★^ p ≤ 0.0001.
(TIF)

S4 Fig Representative IVIS images of bacterial burden in CSM001 challenge mice previously immunized with TMM001. Mice immunized with PBS (A) or with 1.5x10^4^ or 1.5x10^5^ CFU of TMM001 (B) were challenged with CSM001 and imaged for bioluminescence signals at 72 h post challenge and every 7 days thereafter until the experiment end. The intensity of emission is represented as a pseudo-color image.
(TIF)

S5 Fig Gross pathology of TMM001-treated mice. BALB/c mice received either PBS (A) or 1.5 x 10^5^ CFU of TMM001 (B) and at day 21, lungs (A, D), liver (B, E) and spleen (C, F) were extracted and visually assessed for pathological effects. Differences in lungs and livers of both treatment groups were relatively unremarkable. The spleens of TMM001-treated animals were enlarged and contained one or multiple abscesses.
(TIF)

S6 Fig Representative images of immunized and CSM001-infected mice organ pathology. Figures A-L display the types of pathology seen in H&E-stained lungs (A, D, G and J), liver (B, E, H and K), and spleen (C, F, I, L) of CSM001 (1.5 x 10^5^ CFU) challenged BALB/c mice previously immunized with PBS (A-C, G-I) or 1.5 x 10^4^ CFU of TMM001 (D-F, J-L) at 0 h and 48 h post challenge.
(TIF)

S1 Table Diameter (mm) of *B*. *mallei* wild-type and TMM001 colonial growth utilizing individual iron sources. (DOC)
