# Supplementary figures and images for: Characterization of the Burkholderia mallei tonB Mutant and Its Potential as a Backbone Strain for Vaccine Development
Source: PLoS Negl Trop Dis. 2015 Jun 26;9(6):e0003863. doi: 10.1371/journal.pntd.0003863 (PMC4482651; doi:10.1371/journal.pntd.0003863)

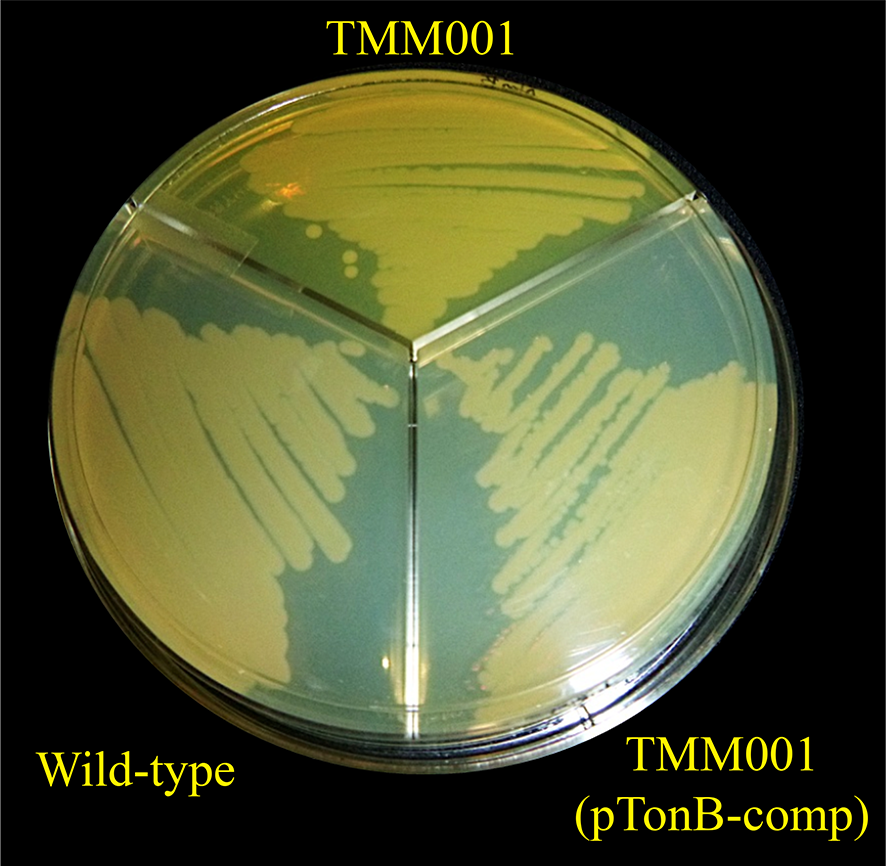

Supplement: S1 Fig — Wild-type (lower left), TMM001 (top) and the complemented ΔtonB (pTonB-comp) (lower right) were grown on LBG + 200 μM FeSO4 for 3 days at 37°C. The figure shows the differences in colony color and modification to the agar media as result of secretion of a pigment by TMM001. (TIF) [file pntd.0003863.s001.tif]

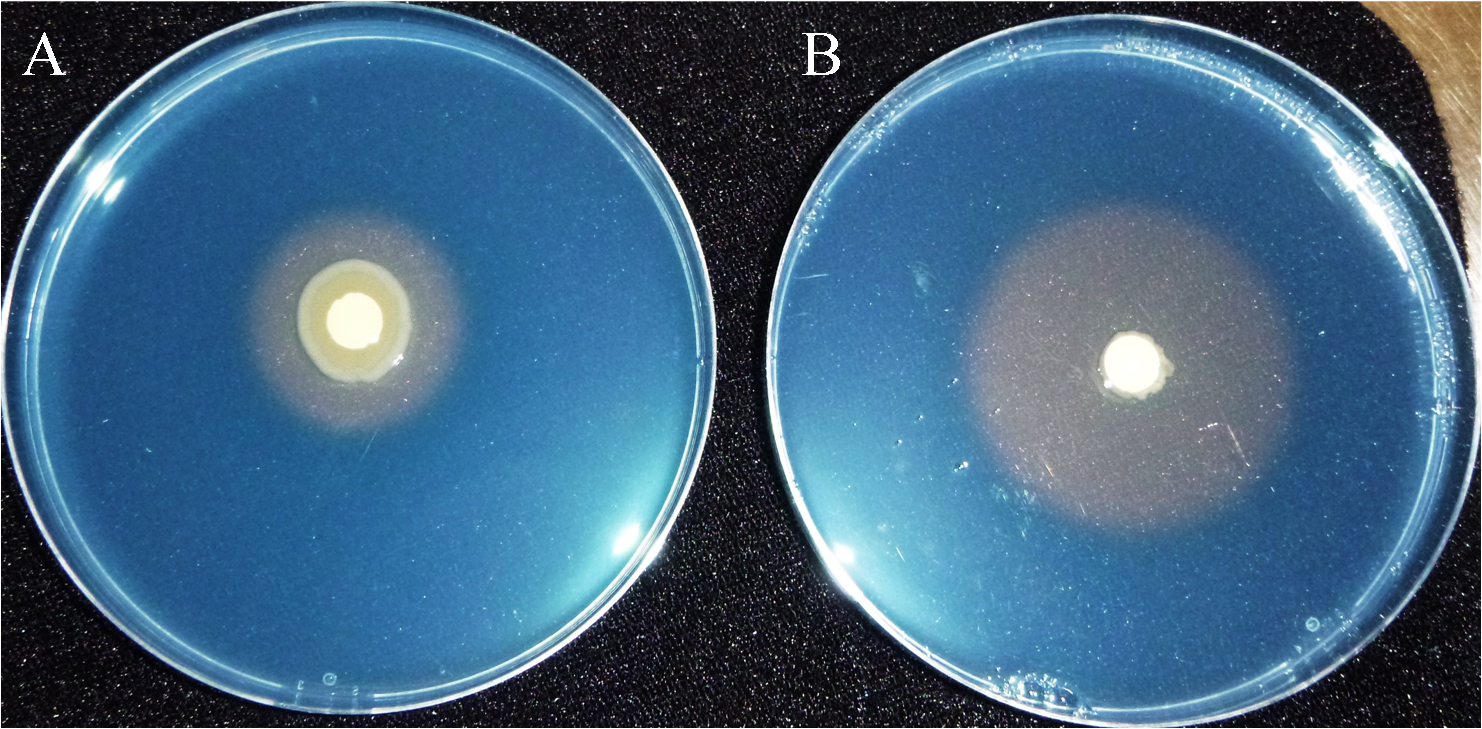

Supplement: S2 Fig — Ten microliters of wild-type (A) and TMM001 (B) overnight cultures grown in LBG were spotted on a filter disk placed on CAS agar media. CAS agar plates were then incubated for at 37°C for 96 h. (TIF) [file pntd.0003863.s002.tif]

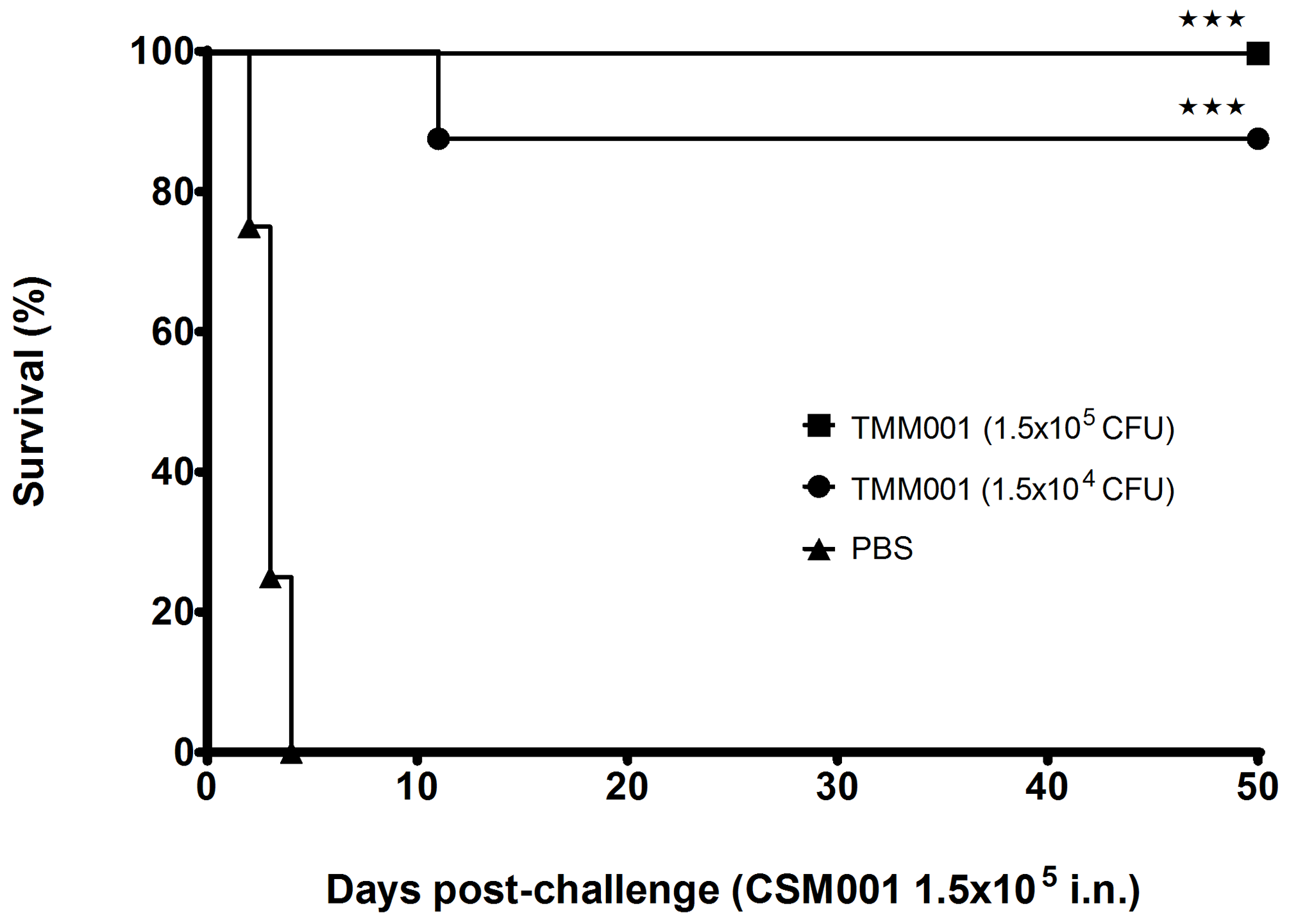

Supplement: S3 Fig — Mice (n = 8) were immunized i.n. with PBS (solid triangle), 1.5 x 10^4^ CFU (solid circle) or 1.5 x 10^5^ CFU (solid diamond) of TMM001. Three weeks later, BALB/c mice were challenged with 1.5 x 10^5^ CFU of a B. mallei reporter strain CSM001. The statistical significance in the different survival times was determined by plotting Kaplan-Meier curves, followed by a log rank test. ★★★★ p ≤ 0.0001. (TIF) [file pntd.0003863.s003.tif]

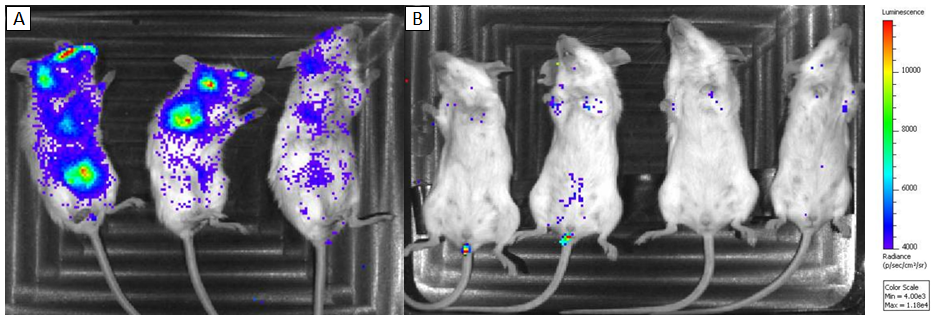

Supplement: S4 Fig — Mice immunized with PBS (A) or with 1.5x10^4^ or 1.5x10^5^ CFU of TMM001 (B) were challenged with CSM001 and imaged for bioluminescence signals at 72 h post challenge and every 7 days thereafter until the experiment end. The intensity of emission is represented as a pseudo-color image. (TIF) [file pntd.0003863.s004.tif]

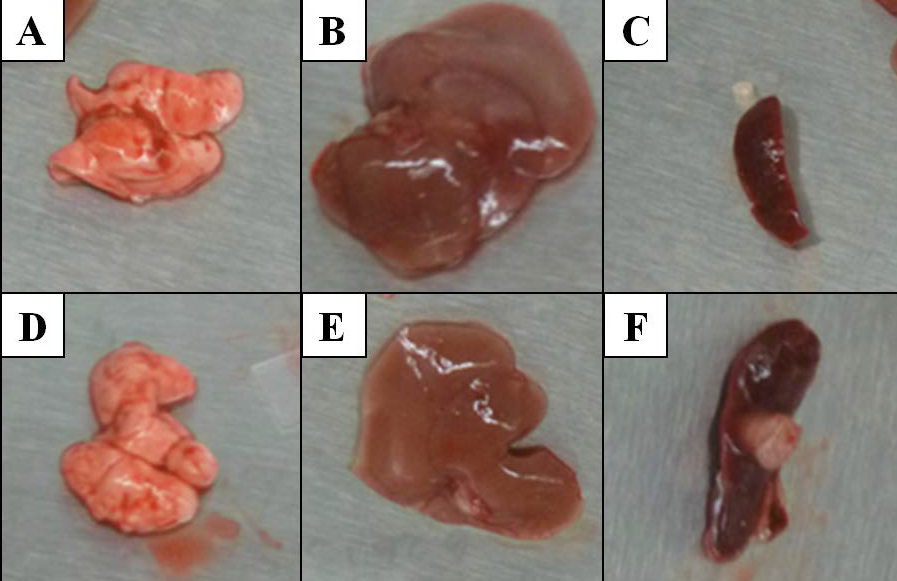

Supplement: S5 Fig — BALB/c mice received either PBS (A) or 1.5 x 10^5^ CFU of TMM001 (B) and at day 21, lungs (A, D), liver (B, E) and spleen (C, F) were extracted and visually assessed for pathological effects. Differences in lungs and livers of both treatment groups were relatively unremarkable. The spleens of TMM001-treated animals were enlarged and contained one or multiple abscesses. (TIF) [file pntd.0003863.s005.tif]

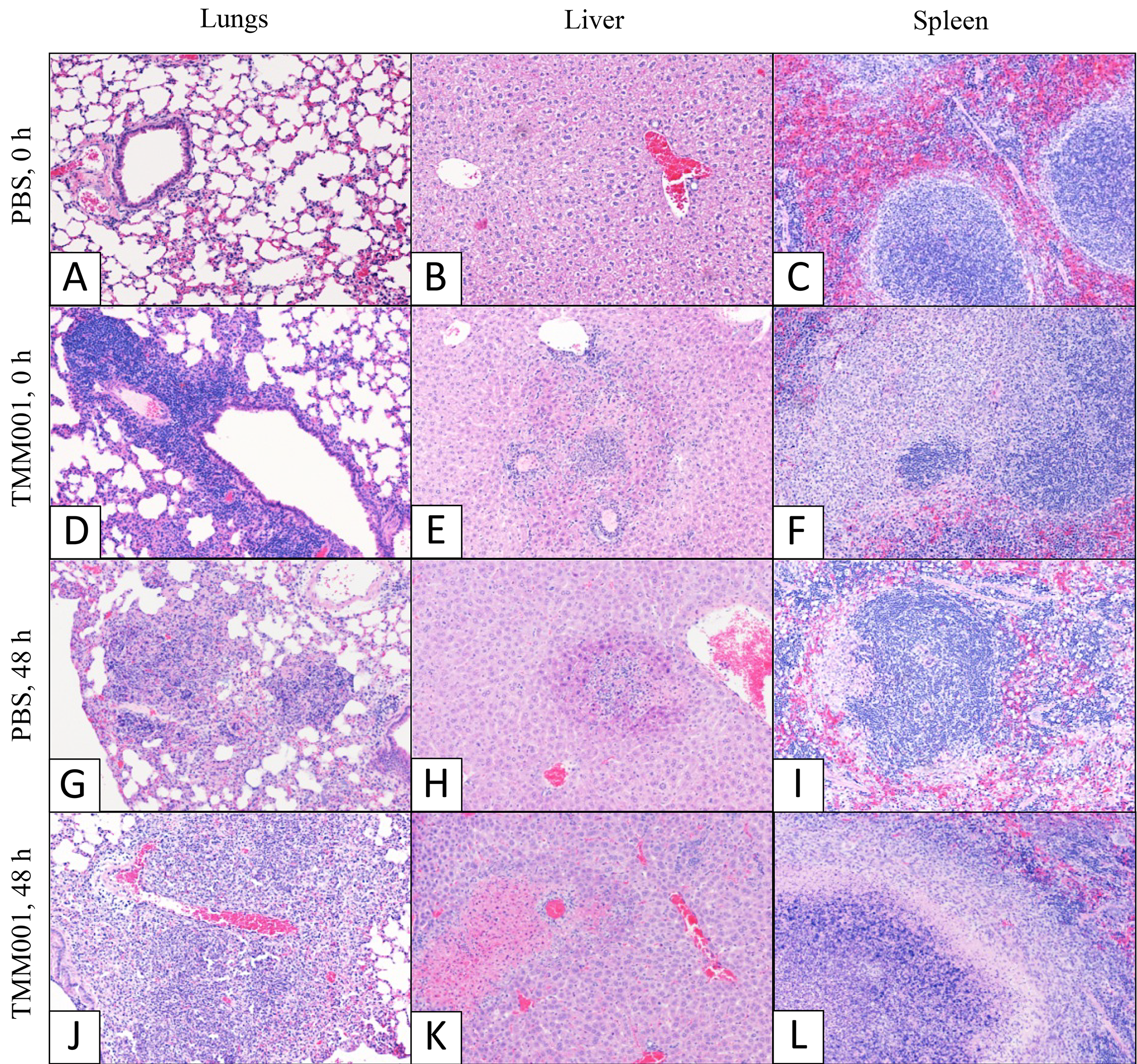

Supplement: S6 Fig — Figures A-L display the types of pathology seen in H&E-stained lungs (A, D, G and J), liver (B, E, H and K), and spleen (C, F, I, L) of CSM001 (1.5 x 10^5^ CFU) challenged BALB/c mice previously immunized with PBS (A-C, G-I) or 1.5 x 10^4^ CFU of TMM001 (D-F, J-L) at 0 h and 48 h post challenge. (TIF) [file pntd.0003863.s006.tif]
