# Supplementary material for: Characterization of the Burkholderia mallei tonB Mutant and Its Potential as a Backbone Strain for Vaccine Development
Source: PLoS Negl Trop Dis. 2015 Jun 26;9(6):e0003863. doi: 10.1371/journal.pntd.0003863 (PMC4482651; doi:10.1371/journal.pntd.0003863)
Supplement: S1 Table — (DOC) [file pntd.0003863.s007.doc]

**S1 Table.** Diameter (mm) of *B. mallei* wild-type and TMM001 colonial growth utilizing individual iron sources.

| **Strain** | **FeSO_4_** | **Hemoglobin** | **Hemin** | **Lactoferrin** | **Transferrin** |
| --- | --- | --- | --- | --- | --- |
| Wild-type | 25.8 ± 3.5 | 14.2 ± 1.8 | 17.7 ± 1.5 | 12.5 ± 0.5 | 12.8 ± 2.1 |
| TMM001 | 14.7 ± 1.5 | 0 | 0 | 0 | 0 |
